# Bone Mineral Density as an Individual Prognostic Biomarker in Patients with Surgically-Treated Brain Metastasis from Lung Cancer (NSCLC)

**DOI:** 10.3390/cancers14194633

**Published:** 2022-09-24

**Authors:** Inja Ilic, Anna-Laura Potthoff, Valeri Borger, Muriel Heimann, Daniel Paech, Frank Anton Giordano, Leonard Christopher Schmeel, Alexander Radbruch, Patrick Schuss, Niklas Schäfer, Ulrich Herrlinger, Hartmut Vatter, Asadeh Lakghomi, Matthias Schneider

**Affiliations:** 1 Department of Neurosurgery, University Hospital Bonn, 53127 Bonn, Germany; 2 Department of Neuroradiology, University Hospital Bonn, 53127 Bonn, Germany; 3 Department of Radiation Oncology, University Hospital Bonn, 53127 Bonn, Germany; 4 Department of Neurosurgery, BG Klinikum Unfallkrankenhaus Berlin, 12683 Berlin, Germany; 5 Division of Clinical Neurooncology, Department of Neurology, University Hospital Bonn, 53127 Bonn, Germany

**Keywords:** lung cancer, surgery for brain metastasis, bone mineral density, survival

## Abstract

**Simple Summary:**

Bone mineral density (BMD) has been shown to be a relevant imaging biomarker for various chronic debilitating diseases. The present study investigated the prognostic value of preoperative BMD values in patients with surgically-treated brain metastasis (BM) related to non-small-cell lung cancer (NSCLC). BMD values were measured in the first lumbar vertebra (L1) in preoperative CT scans and referenced to age-adjusted reference values. Pathologic BMDs were found to exhibit an impaired median overall survival and increased 1-year mortality in the study cohort, and could therefore aid as easily accessible and relevant biomarkers for prognostic assessment and treatment guidance.

**Abstract:**

Patients with BM are in advanced stages of systemic cancer, which may translate into significant alterations of body composition biomarkers, such as BMD. The present study investigated the prognostic value of BMD on overall survival (OS) of 95 patients with surgically-treated BM related to NSCLC. All patients were treated in a large tertiary care neuro-oncological center between 2013 and 2018. Preoperative BMD was determined from the first lumbar vertebrae (L1) from routine preoperative staging computed tomography (CT) scans. Results were stratified into pathologic and physiologic values according to recently published normative reference ranges and correlated with survival parameters. Median preoperative L1-BMD was 99 Hounsfield units (HU) (IQR 74–195) compared to 140 HU (IQR 113–159) for patients with pathological and physiologic BMD (*p* = 0.03), with a median OS of 6 versus 15 months (*p* = 0.002). Multivariable analysis revealed pathologic BMD as an independent prognostic predictor for increased 1-year mortality (*p* = 0.03, OR 0.5, 95% CI 0.2–1.0). The present study suggests that decreased preoperative BMD values may represent a previously unrecognized negative prognostic factor in patients of BM requiring surgery for NSCLC. Based on guideline-adherent preoperative staging, BMD may prove to be a highly individualized, readily available biomarker for prognostic assessment and treatment guidance in affected patients.

## 1. Introduction

Lung cancer remains the leading cause of cancer-related mortality in western nations [1]. More than 50% of patients with non-small-cell lung cancer (NSCLC) present with metastases at the time of diagnosis, including 20–30% with brain metastases (BM) [2,3]. Treatment of patients with NSCLC and BM involves a multimodal and interdisciplinary strategy. Neurosurgical resection of BM is a major component of this process. Nevertheless, determining the optimal therapeutic strategy is a complex matter and varies greatly depending on the patient’s clinical condition and physical integrity. A multidisciplinary assessment of the patient remains therefore imperative [3]. Thus, it appears crucial to identify suitable risk factors that permit a refined treatment stratification of affected patients. In this regard, biomarkers have recently gained importance, mapping not only the consumptive nature of the metastatic cancer itself but also the potential impact of previous treatment [4]. It seems mandatory to detect further predictive biomarkers in order to achieve a more individualized and tailored treatment composition for patients.

The predictive value of body composition imaging markers has already been identified and validated for a variety of cancer types [5,6,7]. Predominantly, sarcopenia and body mass index (BMI) have been identified as relevant risk factors for patients with BM [4,8,9,10]. In addition to muscle mass and BMI, bone mineral density (BMD) has recently been described as another potentially valuable imaging marker in various clinical domains [11,12,13,14]. Furthermore, a potential predictive value of BMD has also been demonstrated in some cancer entities, such as colon and breast cancer, as well as hepatocellular carcinoma and intrahepatic cholangiocarcinoma [15,16,17,18]. As patients with metastatic cancer usually receive guideline-adherent staging examinations prior to further treatment phases, BMD could be opportunistically extracted from the respective cross-sectional images. Thus, BMD might be used to derive a surrogate parameter for the advanced disease process but also for the effects of a previous, possibly debilitating, systemic therapy.

## 2. Materials and Methods

### 2.1. Patients

All patients who had undergone surgery for BM from histopathologically proven NSCLC at the Neuro-Oncological Center of the University Hospital Bonn during the time period between 2013 and 2018 were identified from the hospital information system using a standardized query. Preoperative imaging of the first lumbar vertebral body was required to determine BMD. Therefore, only patients who had received appropriate preoperative staging computed tomography (CT, with depicting of the first lumbar vertebral body) were included in further analysis. Additional data, including patient characteristics, neurological status on admission and during treatment, and radiological findings, were assessed pseudonymously and included in a computerized database (SPSS, version 27, IBM Corp., Armonk, NY, USA). The neurological functional status was monitored based on the Karnofsky performance status (KPS) and categorized into two groups: KPS ≥ 70 versus KPS < 70 as previously described [19]. The age-adjusted Charlson comorbidity index (CCI) and the American Society of Anesthesiologists (ASA) score were additionally obtained to achieve more accurate and precise information about the patients’ preoperative comorbidity burden. Treatment decision was taken by weekly institutional interdisciplinary tumor advisory board meetings for the central nervous system, as previously described [20,21].

Overall survival (OS) was defined as the period of time from the day of BM surgery until death or last observation. All patients for whom follow-up data were unavailable after discharge were ruled out from further analysis.

### 2.2. CT Image Acquisition

CT scans were performed ≤ 2 months prior to the resection of the BM on a Philips- multi-detector scanner at a constant peak voltage of 120 kV with variable protocol-specific tube current (mA) settings. Of note, kV- settings have a strong effect on bony HU values, whereas mA only affect noise levels and not HU values. In all CT examinations, weight-based intravenous contrast was administered, which has a small but measurable effect on trabecular HU values. The CT scanner was calibrated daily for quality control throughout the study to ensure reproducible attenuation figures. As measurements of BMD in low-dose studies might possibly be inaccurate, they were generally excluded from this study.

### 2.3. Image Analysis

A board-certified radiologist (AL) measured mean L1 trabecular attenuation on a single axial CT image in the appropriate plane by manually placing an oval region of interest (ROI) in trabecular space in the anterior whorl body to measure the average attenuation value, as described by Jang et al. [22]. Focal sclerotic or lytic lesions, focal abnormalities, and artifacts were avoided. The patient was excluded if reliable L1 trabecular measurement was not possible. This measurement is also acceptable in the sagittal view. However, in our study, we used only the transverse plane for L1 measurements. In case of compression fracture in L1, either T12 or L2 was used for trabecular attenuation measurement. Image analysis and measurements were all performed using our institutional picture archiving and communication system (PACS). The L1 vertebral level was determined to be the optimal target for opportunistic screening because it is easily identified and captured on all CT scans of the abdomen and chest and is usually defined as the first non-rib-bearing vertebra, which we defined as the vertebral body in this study.

According to the results of Jang et al. [22], patients were subdivided into two different categories based on sex- and age-related normative BMD values. Based on the established range of BMD values defined by Jang et al. [22], the patients were correspondingly divided into subgroups: pathologic versus physiologic BMD.

**Figure 1:**
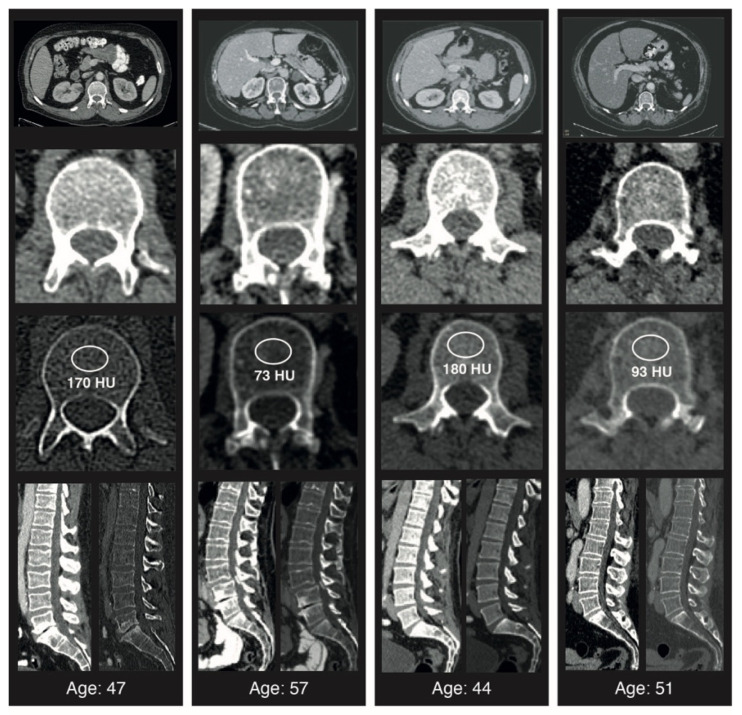
Examples of trabecular L1 attenuation assessment at CT. Transverse (axial) CT scans at the L1 level in adult patients of varying ages and with different average Hounsfield units at the time of diagnosis. Standard placement of the region of interest (ROI) for trabecular attenuation measurement and the mean Hounsfield unit value within the ROI are shown. Sagittal reconstructions with soft tissue and bone windows are shown.

Throughout data acquisition, the radiologist was blinded to all patient characteristics, treatment history, and OS. Figure 1 illustrates exemplary measurements in patients with high and low BMD.

### 2.4. Statistics

Data analyses were performed using the computer software package SPSS (version 27, IBM Corp., Armonk, NY, USA) and GraphPad PRISM (Graphpad Software version 5.0, Inc., San Diego, CA, USA). Categorical variables were analyzed in contingency tables using Fisher’s exact test. The Mann–Whitney U test was chosen to compare continuous variables as the data were mostly not normally distributed. Results with *p* < 0.05 were considered statistically significant. OS was analyzed by the Kaplan–Meier method using the Gehan–Breslow–Wilcoxon test. In addition, a multivariable Cox regression model was applied to determine independent variables for OS.

## 3. Results

### 3.1. Patient Baseline Characteristics

Among the 154 patients with histopathologically proven BM from NSCLC, 59 were excluded due to lack of adequate preoperative CT images showing L1 vertebra or upon lost to follow-up. A total of 95 patients fulfilled the inclusion requirements and were therefore selected for further analysis. The median age at admission was 63 years (IQR 58–50 years). The gender distribution was balanced with 47 (49%) women and 48 men (51%). Thirty-nine patients (41%) presented with multiple BMs at the time of surgery. Patients revealed a preoperative KPS ≥ 70 in a total of 87% of cases (*n* = 83). None of the patients suffered from spinal metastases at the time of surgery. Median BMD was 125 HU (IQR 95–165 HU). The median OS for all patients with surgically treated BM was 9 months (range 3–24 months). Detailed baseline patient characteristics are shown in Table 1.

### 3.2. Patient- and Disease-Related Characteristics Dependent on Physiologic and Pathologic Bone Mineral Density-Levels

Forty-nine of 95 patients with surgically-treated BM from NSCLC (52%) exhibited physiologic preoperative BMD-levels with a median BMD of 140 HU (IQR 113–159 HU). Compared with this, 46 of 95 patients (48%) revealed pathologic preoperative BMD measurements with a median BMD of 99 HU (IQR 74–195 HU, *p* = 0.03). Multiple BMs were significantly more frequent in patients with pathologic compared to physiologic BMD levels (56% versus 26%; *p* = 0.004). Both groups of patients with physiologic and pathologic BMD levels did not significantly differ regarding age at admission, sex, comorbidity burden objectified by the CCI-index, and preoperative KPS. Further stratification according to baseline characteristics is given in Table 2.

### 3.3. Influence of Pathologic Bone Mineral Density on 1-Year Mortality and Overall Survival

**Figure 2:**
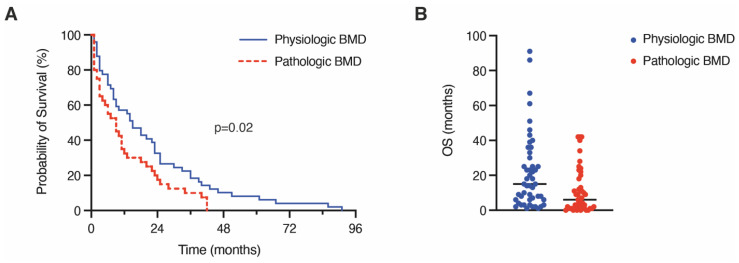
Kaplan–Meier survival curves (**A**) and scatterplot (**B**) depicting OS dependent on pathologic versus physiologic BMD levels. BM, brain metastasis; BMD, bone mineral density.

Mortality rate analysis indicated a significantly increased likelihood of death in relation to pathological BMD (1-year mortality rate physiologic BMD: 43%; pathologic BMD 72%; Table 2). The median OS in patients with preoperatively determined pathological BMD was 6 months compared to 15 months in patients with physiological BMD (*p* = 0.002; Table 2, Figure 2A,B).

We conducted a multivariable logistic regression analysis in order to identify independent predictors of 1-year mortality in patients that had undergone surgery for BM from NSCLC. The multivariable analysis identified “multiple BM” (*p* = 0.01, OR 0.5, 95% CI 0.1–0.7) and “pathologic BMD” (*p* = 0.03, OR 0.5, 95% CI 0.2–1.0) as significant and independent predictors of 1-year mortality (Nagelkerke’s R2 0.2).

## 4. Discussion

The impact of body composition imaging markers is becoming increasingly relevant in the field of oncology [5,6,7]. A major benefit of imaging markers lies within the accessibility of their kind. Each patient undergoes routine staging examinations during screening and/or follow-up care. From these scans, a variety of information can be harvested opportunistically, thus yielding additional prognostic value for individual patient care [23].

One of the most thoroughly researched imaging markers of the last decade is the quantification of skeletal muscle mass in various imaging modalities. When being applied as a surrogate parameter for sarcopenia, a condition of pathologically diminished muscle mass, it has proven valuable for numerous oncological conditions, mainly serving as a predictor for OS or tumor response assessment [4,8,24,25]. For instance, the prognostic significance of low muscle mass has been linked to a poorer OS in patients with BM in NSCLC [4]. In addition to this, physical strength is a relevant clinical parameter which complements well with quantified muscle mass and has therefore also been investigated for multimodal oncological surveillance [26]. Nevertheless, an independent and specific assessment of physical capabilities is often challenging, as they are managed slightly differently by various treatment centers and are not retrospectively accessible.

BMD constitutes another imaging marker that recently has been shown to provide valuable prognostic insights for various severe illnesses. among them cardiovascular diseases and chronic lung pathologies [13,27,28]. The present study investigates the utility of BMD as a prognostic imaging marker in patients with surgically resected BM from NSCLC. We found pathologically decreased BMD values to correlate to elevated 1-year mortality rates and worsened OS. Demographic data and previous illnesses were excluded as confounders, as no relevant differences were found between the groups. Thus, in the present patient population, discriminatory power based on bone density can be assumed to be evident. Cancer patients are known to be at a particularly elevated risk of reduced BMD and osteoporosis. A large Danish registry study found that osteoporosis was linked to increased risk of NSCLC occurrence for both men and women younger than 70 years [29]. Though the exact underlying pathophysiological mechanisms of osteoporosis in NSCLC patients is far from understood, tumor-induced metabolic and hormonal changes might partly add to this correlation. Along these lines, about half of patients with NSCLC exhibit a positive estrogen receptor status [30] suggesting NSCLC to interfere within these hormonal circuits. Further, it is important to be aware that the present study cohort was comprised of patients with BM that were at advanced stages of metastatic NSCLC disease. Here, preceding therapy regimes might significantly impact on BMD levels via secondary-induced vitamin D deficiency, long-lasting corticosteroid application, nutritional differences, and lifestyle changes [31,32,33]. Furthermore, the potential predictive value of BMD has also been demonstrated other cancer entities, such as colon and breast cancer, as well as hepatocellular carcinoma and intrahepatic cholangiocarcinoma [15,16,17,18]. It has also been found that both increased and decreased bone density may both be possible risk factors for various types of cancer [34]. However, definite causations for these observed correlations remain unclear and need to be further investigated.

One might speculate whether the observed correlation between BMD and survival would also entail therapeutic implications. Knowledge of mortality reduction via bisphosphonate application in patients with osteoporosis [35] would imply potential survival benefits in NSCLC patients with BM that are treated with drugs interfering in bone metabolism. The fact that BMD as a novel imaging biomarker can be efficiently extracted from existing staging exams makes it a promising candidate for studies based on our results. A pragmatic approach would be to automate the extraction of BMD in staging examinations of oncological patients, e.g., by training a dedicated AI. This would allow BMD to be seamlessly implemented in scoring systems with other clinical parameters.

Nevertheless, further multicenter studies are needed to more comprehensively evaluate any potential prognostic impact of reduced BMD in cancer patients and to delineate potential therapeutic implications. Nonetheless, the results of the present study suggest that BMD might serve as highly individual biomarker for the prognostic consequences of the consuming cancer disease, its previous treatment and/or the patient-specific preoperative physique.

### Limitations

The present study has several limitations. The first is the retrospective nature of the study with the risk of potential unmasked selection bias. Even though the size of the cohort was rather limited, due to careful selection criteria the two investigated subgroups were comparable with regards to the most relevant characteristics such as gender, age, and preoperative KPS, which to a certain extent compensates for this limitation. However, future multicenter studies are needed in order to sufficiently cope with the small study population of the present manuscript. The assessment of the L1 BMD is not yet a clinically accepted method for bone density measurement. However, BMD measurement was performed in accordance to a recently published report by Jang et al. [22] where normative values were obtained in 20,000 patients, so that there are at least reliable, scientifically validated and promising results for the method.

## 5. Conclusions

The present study suggests preoperative L1 BMD values to constitute a previously unrecognized prognostic factor and therefore an imaging biomarker in NSCLC patients undergoing BM surgery. Based on guideline-appropriate preoperative staging in patients with metastatic cancer, BMD may prove to be a highly individualized, readily available biomarker for prognostic assessment, treatment guidance, and counseling of affected patients with BM due to NSCLC. In order to investigate the specific meaning of BMD, larger, multicenter and prospective studies are required, which may further include other types of cancer.

## Figures and Tables

**Table 1:** Baseline characteristics *.

No. of Patients	*n* = 95
Median age (IQR) (in y)	63 (58–70)
Female sex	47 (49)
Multiple BM	39 (41)
Preoperative KPS ≥ 70	83 (87)
Median CCI-index (IQR)	9 (8–9)
ASA ≥ 3	49 (51)
Median BMD (in HU)	125 (95–165)
1-year mortality	54 (57)
Median OS (in months)	9 (3–24)

* Values represent number of patients unless indicated otherwise (%). ASA, American Society of Anesthesiology physical status classification system; BM, brain metastasis; BMD, bone mineral density; CCI, Charlson comorbidity index; HU, Hounsfield unit; IQR, interquartile range; KPS, Karnofsky performance status; y, years.

**Table 2:** Patient- and disease-related characteristics dependent on the presence of physiologic and pathologic BMD-levels *.

	Patients with Physiologic BMD *n*= 49	Patients with Pathologic BMD *n*= 46	*p*-Value
**Median BMD (HU, IQR)**	140 (113–159)	99 (74–195)	**0.03**
**Median Age (yrs, IQR)**	64 (58–71)	62 (57–70)	0.59
**Female sex**	24 (49)	23 (50)	1.0
**Multiple BM**	13 (26)	26 (56)	**0.004**
**Preoperative KPS ≥ 70**	44 (90)	39 (85)	0.54
**Median CCI-index (IQR)**	9 (8–9)	9 (8–10)	0.46
**Preoperative radiotherapy**	1 (2)	1 (2)	1.0
**1-year mortality**	21 (43)	33 (72)	**0.007**
**Median OS (in months)**	15 (6–32)	6 (1–19)	**0.002**

* Values represent the number of patients unless indicated otherwise (%). BM, brain metastasis; BMD, bone mineral density; CCI, Charlson comorbidity index; IQR, interquartile range; HU, Hounsfield unit; KPS, Karnofsky performance status; OS, overall survival; yrs, years.

## Data Availability

The authors confirm that the data supporting the findings of this study are available within the article.
